# Use of Air-Protected Headspace to Prevent Yeast Film Formation on the Brine of Leccino and Taggiasca Black Table Olives Processed in Industrial-Scale Plastic Barrels

**DOI:** 10.3390/foods9070941

**Published:** 2020-07-16

**Authors:** Gino Ciafardini, Biagi Angelo Zullo

**Affiliations:** Department of Agricultural, Environmental and Food Sciences, University of Molise, Via De Sanctis, I-86100 Campobasso, Italy; ciafardi@unimol.it

**Keywords:** Leccino, plastic barrels, table olives, Taggiasca, yeast film

## Abstract

The formation of yeast film on the brine of black table olives during fermentation in plastic barrels on an industrial-scale could be critical for the quality of the product. In order to prevent the formation of yeast film on the brine surface, a structural modified industrial barrel, which excludes oxygen from the headspace, was tested. Tests carried out during two years indicated that the yeast film contamination reached the maximum values at eight months of fermentation, equal to 19% and 24% respectively, for the Taggiasca and Leccino olives, processed in unmodified industrial plastic barrels. No yeast films formed on brines from the same varieties of olives processed in the modified plastic barrels. The brines of both varieties of olives processed in the industrial barrels displayed three dominant yeast species, while five species were detected in the brines from the modified barrels. *Wickerhamomyces anomalus* and *Pichia manshurica* were the main producers of yeast films. However, *P. manshurica* unlike the other yeasts, has shown also a biotype unable to produce films on the brine of the olives. The brines of Leccino and Taggiasca processed in the modified barrels, compared to the control, showed a higher titratable acidity and a higher concentration of CO_2_ useful to prevent the yeast film formation.

## 1. Introduction

Table olives are the most important fermented vegetables in the Mediterranean area due to their global economic importance. Their production exceeded 2.9 million tons in the 2019/2020 season. The main producers are Spain, Egypt, Turkey, Algeria, Italy, Greece, and Portugal [1]. The production of table olives is increasing in other locales, such as South America, Australia, and the Middle East [1]. Olives cannot be consumed directly from the tree due to the presence of compounds like oleuropein, which is a bitter phenolic glucoside consisting of glucose, elenolic acid, and hydroxytyrosol compounds [2]. The oleuropein content in olives is influenced by various factors that include the variety and degree of ripeness at the time of harvest [3,4,5]. The processing systems of table olives include the Sevillian and Californian methods, which involve the chemical treatment of the fruits and the natural fermentation of the olives in brine without pre-treatment with chemical products. These approaches are also termed natural black table olive processing systems [6]. During the debittering process, natural black table olives are subjected to spontaneous fermentation involving a microbial community, including members of lactic acid bacteria (LAB) and yeasts. The salt concentration and the phenol content in the brine affect the dominance of LAB and yeast during the fermentation period [7]. High phenolic content and a salt concentration greater than 10% are associated with brine acidification with citric acid, which favor yeasts during the fermentation period [8,9,10]. The natural black table olives processing system applied to the Leccino and Taggiasca varieties is the main traditional debittering system used in different areas of Italy. The Leccino variety is cultivated in different areas, whereas the Taggiasca variety is typical of the Liguria region (North Italy), where it is cultivated for the production of both oil and table olives. To produce traditional natural fermented table olives, the fruits are harvested when they become black. They are sorted, rinsed with water, and placed in 160-200-L polyethylene (PE) or polyvinylchloride (PVC) plastic barrels. The barrels are filled with freshly prepared brine with a salt concentration of 12% (*w*/*v*), acidified with the lactic or citric acid, sealed and stored at room temperature in sheds to protect from the sun and outdoor conditions. The fruits marinade in the brine until they lose their bitter taste. After six or more months, they are placed in jars, filled with fresh brine, and pasteurized. Since this method of processing is characterized by a high salt concentration of the brine, it is often referred to as Greek-style processing. The 200 L barrels normally used in the processing of natural black table olives include a barrel with a flat lid locked to the rest of the barrel by a threaded crown, and a second type having a slightly conical lid and a threaded cap. The second type allows to check and added fresh brine. Among these two commercial prototypes, the second type is often preferred because it can eliminate the residual air under the lid by adding new brine. To allow a correct fermentation of the olives in brine, in addition to ensuring a sufficient concentration of salt and a pH lower than 4.30 in the brine, it is also necessary to ensure the anaerobic conditions inside the plastic barrels. Under anaerobic conditions, fermentation is spontaneous and begins as soon as the olives are pickled. LABs dominate in green olive brines, while mainly fermentative yeasts including *Saccharomyces cerevisiae* are found in black olive brines [11]. Entry of the outside air must be avoided. The penetration of air into the barrels may occur if the integrity and tightness of the rubber gaskets located under the covers are suboptimal, and because of day–night temperature variations. The hot daytime hours can cause dilatation of the contents of barrels, which can push some of the brine out of the containers. In contrast, the cooling of the mass during the night can create a certain vacuum in the barrels that draws external air in the interspace between the lid and the brine surface. This behavior is very pronounced in cases where the barrels are stored under canopies or outdoors, and during transport from one location to another. The presence of oxygen in the headspace of plastic barrels favors the formation of microbial films on the surface of the brine. The films, also known as yeast kahm or flor, consist of an aggregation of oxidative yeasts, molds, bacteria and polymers [12]. Formation of films of molds and yeasts on the surface of brine is a spoilage factor, since it can worsen the quality of the product. Oxidative yeasts residing in the biofilms can metabolized lactic and acetic acid under aerobic conditions, reducing brine acidity and increasing pH, which can allow the growth of others undesirable microorganisms with the consequent loss of product quality and reduced food safety [13,14]. In addition, the microbial film grown in the brines in presence of air close to the headspace of the barrels produces enzymes that are responsible for the sweetening of the olives, serious sensorial defects, and the accumulation of mycotoxins harmful to human health [15,16,17]. The formation of microbial films, including yeast films, on the brine is a serious problem for many processing companies that use plastic barrels. The formation of microbial films can be inhibited by chemical treatments or physically. One of the chemical treatments used mainly for storing black ripe olives involves the use of sodium benzoate. However, this treatment is not recommended for European processes because of the residual amount of preservative in the final product which is considered a harmful and allergenic agent, not allowed by some countries. On the other hand, the consumption of organic foods is increasing and black olives fit the definition since they are processed without chemical treatment [18]. One of the most common physical remedies consist of remove the air in the headspace through the correct filling of the barrels during fermentation. This intervention is incorporated in the list of actions in the Hazard Analysis and Critical Control Points. However, for many companies that transform black table olives using thousands of plastic barrels, it becomes difficult to control microbial film formation in a timely manner for each barrel. The reality can be the discarding of large batches of altered olives, especially when the plastic barrels do not have a control cap on the lid for visual checks. Considering the lack of research on this topic, the main purpose of this work was to study the appearance of films in the brine surface in industrial barrels and the application of a practical physical system to prevent its appearance.

## 2. Materials and Methods

### 2.1. Design of Experiences

The trials concerning the control of the yeasts film grown on the brine surface during the natural black table olives fermentation of the Leccino and Taggiasca varieties were accomplished in industrial-scale using 200 L plastic barrels. The experiments were performed in two consecutive years at a processing plant for Leccino and Taggiasca olives produced in central and northern Italy, respectively.

#### 2.1.1. Study of Microbial Film Appearance

The aim of the tests carried out in the first year of experimentation was to study the dynamics of the appearance of the microbial film in the industrial plastic barrels during fermentation of Leccino and Taggiasca black table olives. A study on the rate of Leccino and Taggiasca black table olive brine contamination with yeast film was carried out during the first year of investigation at the processing site. A total of 200 industrial PVC (polyvinylchloride) barrels were divided into two groups, with 100 barrels each for Leccino and Taggiasca olives. The Leccino and Taggiasca table olives from central and northern Italy, respectively, were harvested in November when the maturation phase was suitable for processing. They were then washed with water, classified, and poured into 200 L PVC barrels filled with 120 kg of olives and 80 L of brine containing 12% (*w*/*v*) NaCl, and then acidified with 0.3% (*w*/*v*) citric acid. At the time of preparation, the barrels were filled completely with brine, closed with a full-sized lid provided with a cap for visual checks and stored at room temperature in a shed for 10 months. During the fermentation period, the level of the brine was checked every month in all barrels. When necessary, proper topping-up with fresh brine was performed. Every 2 months, the visual check was performed on all barrels of both varieties of olives, and the number of barrels contaminated by yeast film were recorded. Each yeast film was skimmed off of the brine surface and the barrels were proper topped-up with fresh brine.

#### 2.1.2. Microscopy Observation of the Microbial Films

Scanning electron microscope (SEM) observations were performed using the microbial film collected on the brine surface stored in barrels contaminated with the yeast film during the first year of study described above. The biomasses supported with a nitrocellulose membrane filter with a porosity of 0.45 µm were fixed in 1 mL of 3% glutaraldehyde (*v*/*v*) in 0.1 M phosphate buffer, pH 7.2, for 12 h. The samples were rinsed in the same buffer three times and then dehydrated (twice for each solution) in a graded ethanol series (20%, 40%, 60%, 80%, 95%, and 100%) for 10 min each with a final wash in acetone for a better CO_2_ substitution during the dehydration procedure, at a pressure of 1200 bars. Subsequently, all samples were dried in a model K850 CO_2_ critical point drier (Emitech, Montigny-le-Bretonneux, France) and then covered with palladium gold in a K550 device (Emitech) before SEM observation.

#### 2.1.3. Detection of Microorganism Populations

At the time of testing, 10 barrels each of the Leccino and Taggiasca table olives described above were randomly selected and numbered. During the fermentation, 1000 mL of brine was collected at mid-depth of each labelled barrel using a removable sterile plastic probe connected to a mobile vacuum line. The samples were each poured into a sterile Pyrex sterile glass flask and transported immediately to the laboratory for the microbiological analyses described below. The brine microbiota was evaluated in the samples collected at the beginning of the experiment (November) and after 2, 4, 6, 8, and 10 months of fermentation. The microorganisms analyzed were yeasts, total aerobic bacteria (TAB), LAB (lactic acid bacteria), enterobacteria, and molds [19]. The brine samples were serially diluted by a factor of 10 using sterile Ringer’s solution 0.9% (*w*/*v*). Aliquots of each dilutions were spread on agar media. For the enumeration of total culturable yeasts, serial dilutions of the brines were plated onto a malt yeast glucose peptone (MYGP) agar medium (pH 7) with the following composition (per L distilled water): 3 g of yeast extract (Biolife, Milan, Italy), 3 g of malt extract (BBL, Cockeysville, Maryland, USA), 2.5 g casein bacto tryptone (BD, Sparks, Maryland, USA), 2.5 g of soy peptone (Biolife, Milan, Italy), 10 g of D-glucose (Merck, Germany) [20]. Petri dishes containing MYGP agar medium supplemented with 100 µg/mL of chloramphenicol were inoculated with 0.2 mL of sample and incubated at 30 °C for 5 days. TAB were enumerated after 24 h of incubation at 30 °C on nutrient agar (CM 0003, Oxoid, UK) supplemented with 0.05% (*w*/*v*) cycloheximide to prevent the growth of yeasts. LAB were enumerated on de Man-Rogosa Sharpe (MRS) medium (Biolife, Milan, Italy), containing 0.05% (*w*/*v*) cycloheximide (Sigma-Aldrich, St. Louis, MO, USA) and incubated at 30 °C for 4 days under anaerobic conditions. Enterobacteria were evaluated on Violet Red Bile Glucose agar (VRBGA; Biolife) incubated at 37 °C for 24 h. Molds were evaluated after 7 days of incubation at 28 °C using glucose yeast extract agar (GYEA; Oxoid, Basingstoke, UK). All plates were examined visually for typical colony types and morphological characteristics, which were recorded along with the corresponding growth medium. The results are expressed as log values of colony forming units per mL of brine (Log CFU/mL).

#### 2.1.4. Yeast Isolation and Identification

The yeast colonies obtained from microbiological analysis of the Leccino and Taggiasca brines were used to prepare a series of masters using MYGP agar medium [21]. All the colonies from each analyzed brine sample, were distributed in several masters established in petri dishes containing 100 colonies each. After 3 days of incubation at 30 °C, the masters were replicated on CHROM agar Candida medium (BBL 4354093) and tested as previously described [22]. The masters were prepared in duplicate using small sterile wooden sticks to transfer the single colonies into petri dishes with the two media. The yeast cultures grown on MYGP agar medium were analyzed after 10 days incubation at 30 °C, whereas the corresponding cultures grown on CHROM agar Candida medium were observed after 7 days of incubation at 30 °C, and their chromogenic characteristics were recorded. Yeasts grown on MYGP agar medium were analysed individually by examination using an optical microscope (Olympus, Milan, Italy) to ascertain the presence of pseudohyphae and cell shape and size. The main characteristics suitable for distinguishing the different homogeneous chromogenic yeast groups were cell shaped (round vs. elongated), the presence of pseudohyphae, and colony morphology and color. To assess the predominance rates of the wild yeast species in the brines of the industrial barrels, the entire microbial population was divided into homogeneous groups of yeasts from which the representative yeasts were isolated and identified. From each homogeneous chromogenic yeast group of the Leccino and Taggiasca brine samples, 10 yeast isolates were randomly selected and analysed by sequencing the approximately 600 base-pair D1/D2 region of the large (26S) ribosomal subunit using primers NL1 and NL4 as previously described [23]. The ribosomal sequence obtained from the NL1 primer was compared to those of yeast species in the public gene database using a BLAST search of call GenBank+EMBL+DDBJ+PDB sequence on the NCBI website http://www.ncbi.nlm.nich.gov/blast.

#### 2.1.5. Simulation of Yeast Film Formation

The formation of yeast film in the air–liquid interface was detected using yeast species that were determined in the preceding microbiological analyzes to be predominant in the brine samples of the Leccino and Taggiasca varieties collected from the industrial barrels after 10 months of fermentation. Yeast strains belonging to the species *Saccharomyces cerevisiae*, *Pichia manshurica*, *Wickerhamomyces anomalus*, *Candida boidinii*, and *Zygosaccharomyces mrakii* isolated from the Leccino and Taggiasca brines were tested using the MYGP broth substrate and the same brines of origin, respectively. The test was accomplished in triplicate using Pyrex tubes equipped with a cotton caps and large meshes containing MYGP broth medium and filter-sterilized olive brines. Yeast cultures grown for 2 days at 30 °C were centrifuged at 10,000× *g* (Hettich Instruments, Tüttlingen, Germany) and then re-suspended in sterile distilled water to an optical density at 600 nm (O.D._600_) of approximately 0.7. A total of 100 µL of yeast inoculum was dispensed into the corresponding tubes with 3 mL of MYGP broth medium and sterile Leccino and Taggiasca olive brines. The tubes were stirred for 3 s and then incubated for 15 days at 30 °C. Yeasts that exhibited a microbial film at the air–liquid interface of the tube tests were visually assessed as yeast film producers and recorded.

### 2.2. Studies to prevent the Development of Yeast Films

The second year analyses were designed to assess the ability to prevent the development of yeast films on the surface of the brines using the structurally modified barrel that excluded oxygen from the headspace. The tests were performed using the black table olives of the Leccino and Taggiasca varieties processed using the modified and unmodified industrial 200 L plastic barrels. During the 8-month fermentation period, the brine of each barrel was analyzed to determine yeast film contamination, microbiota composition and physicochemical characteristics.

#### 2.2.1. Tests with Modified and Unmodified barrels

The modified barrels, unlike the others used as control, were jacketed inside with a food grade high-density polyethylene (HDPE) bag appropriate for brining. Each HDPE bag had a diameter of 960 mm and a height of 1500 mm, compatible with the internal measurements of the barrels to be modified. Each HDPE bag was adhered to the internal surface of the barrel by swelling them through the upper opening with a light jet of air produced by a compressor. The upper part of each HDPE bag that exceeded the height of the barrel transiently protruded outside the opening of the same container. The tests were set up using 24 barrels for each variety of olive consisting of 12 modified barrels and 12 unmodified barrels (control). The two types of barrels were divided into three groups with four barrels randomly distributed in a shed of the table olive processing company. After harvesting, the Leccino and Taggiasca table olives were prepared for the same tests using the same procedures described in Section 2.1 for the tests of the previous year. Finally, they were placed in all barrels using 120 kg of fruit and approximately 75 L of brine containing 12% (*w*/*v*) NaCl acidified with 0.3% (*w*/*v*) of citric acid. Subsequently, the upper part of each HDPE bag that exceeded the height of the modified barrel was wrapped to expel all the internal air and then sealed tightly with plastic bands. Successively, all barrels were filled completely with brine, closed with a full-sized lid provided with a cap for visual checks, and stored 8 months at room temperature. During the fermentation period, the brine level was checked every month in all barrels, and correct coverage with brine was carried out. Every 2 months, all barrels of both olive varieties were visually inspected. Those contaminated with yeast film were recorded and then decontaminated as previously described. During the fermentation period, starting from the second month and at 2-month intervals, the brine was collected four times from each barrel for microbiological and chemical analysis. Brine was collected from the modified barrels by opening the upper part of the HDPE bags, which were closed immediately after sampling, and the barrels were refilled with fresh brine, similar to all the others. Microbiological analysis and the yeast film assay were performed with the same procedure described above.

#### 2.2.2. Physicochemical Analysis

##### pH, and Titratable Acidity

The pH of the olive brine was measured periodically using a pH meter with an In Lab Routine Pro probe (Mettler, Toledo, USA). The measurement was performed three times. Titratable acidity assays were performed with 5 mL of brine in 45 mL of distilled water and 20 µL of a 1% phenolphthalein indicator dissolved in isopropanol (Thermo Fisher Scientific, Waltham, MA, USA). NaOH (0.1 N) (Thermo Fisher Scientific) was added until the solution retained a light pink colour (approximately pH 8) for 30 s. Titratable acidity was expressed as g of lactic acid per L. Each type of chemical analysis was repeated three times.

##### Total Phenol Content in Brine

A total of 1.5 mL of each brine was centrifuged at 12,000 × *g* for 5 min and 1 mL supernatant was diluted in 9 mL of distilled water. Subsequently, 0.1 mL of diluted brine was added to a screw cap tube containing 0.9 mL of 0.5 N sodium bicarbonate solution (pH 8.5) and 1 mL of Folin–Ciocalteu’s phenol reagent (Sigma-Aldrich) diluted to 1:10 (*v*/*v*). In addition, a treatment consisting of 0.1 mL of distilled water, 0.9 mL of sodium bicarbonate and 1 mL of Folin–Ciocalteu’s phenol reagent (1:10, *v*:*v*) was used. The colorimetric reaction proceeded with the samples in the dark at room temperature for 2 h. After briefly stirring, the samples were analysed with a model 6300 spectrophotometer at a wavelength of 765 nm (Jenway, Staffordshire, UK). The results are expressed as mg of caffeic acid equivalent per mL of brine. The analysis of total phenols was repeated three times.

##### Free Carbon Dioxide (CO_2_) Content in Brine

The free CO_2_ content in the brine was determined by titration method reported by the American Public Health Association [24] with some modifications. After collection, the brine samples were stored in closed bottles and transported to the laboratory. The analysis was performed by dividing each sample into two 50 mL fractions. The first fraction was immediately analysed, while the second was placed in a beaker and stored overnight at −20 °C. Subsequently, the CO_2_ remaining in the sample was removed by establishing a slight flow of N_2_ gurgle in the brine for 30 min followed immediately by analysis. The brine samples were treated with Na_2_CO_3_ (0.0454 N) (Sigma-Aldrich) until a pH value of 8.30 was reached as judged using a pH meter (Mettler). The free CO_2_ content in brine was calculated by subtracting the free CO_2_ content of untreated brine from that obtained by the treated brine. The free CO_2_ content was expressed as mg CO_2_ per L of brine. The chemical analysis was repeated three times.

### 2.3. Statistical Analysis

Statistical software (ver. 7.0) was used for data processing (statsoft for Windows, Tulsa, OK). Comparisons among means were performed with Duncan’s multiple-range test (one-way ANOVA). Differences were considered significant at *p* < 0.05.

## 3. Results

### 3.1. Contamination of Brine by Microbial Film in Industrial Barrels

**Figure 1:**
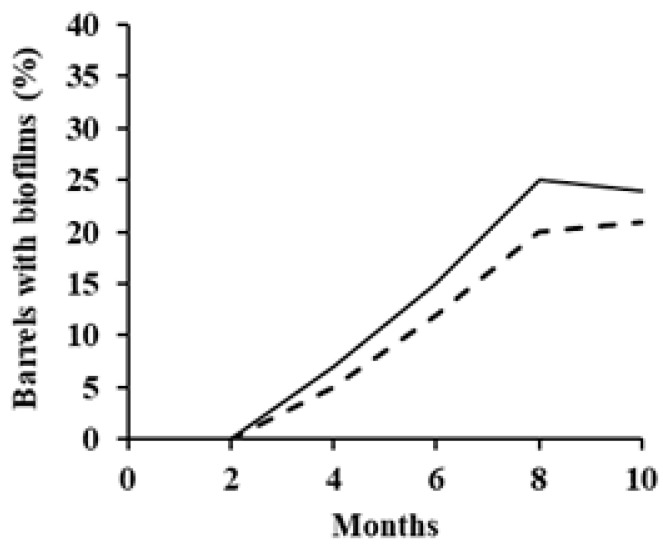
Dynamic of the yeast film contamination in the brine of black table olive stored in plastic barrels in an industrial processing plant (-, Leccino; —Taggiasca).

The tests performed during the first year of investigation on the processing of the Leccino and Taggiasca black table olives in 200 L industrial plastic barrels were designed to detect the contamination of the brines with microbial film. The appearance of the microbial film on the brine surface of both varieties of olives was not evident in the first two months of fermentation. In subsequent months, the number of the contaminated industrial barrels increased progressively until reaching the maximum values at eight months of fermentation, equal to 19% and 24%, respectively, for the Taggiasca and Leccino olives. However, after eight months of fermentation, the number of contaminated industrial barrels tended to be lower than in the previous period (Figure 1).

**Figure 2:**
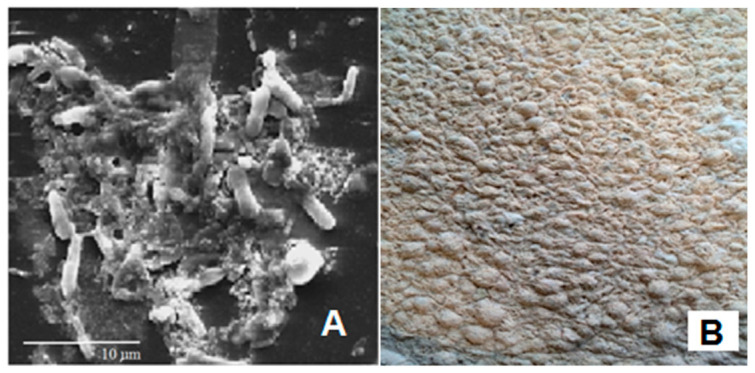
SEM observation of a representative microbial film (**A**) from brine sampled in the headspace of a barrel contaminated by yeast film (**B**).

SEM observation and direct visual analyses of the brines indicated that the microbial film that appeared on the brine surface of the contaminated barrels was mainly made up of yeasts (Figure 2).

#### 3.1.1. Microbiological Analysis and Yeast Species Isolation

**Figure 3:**
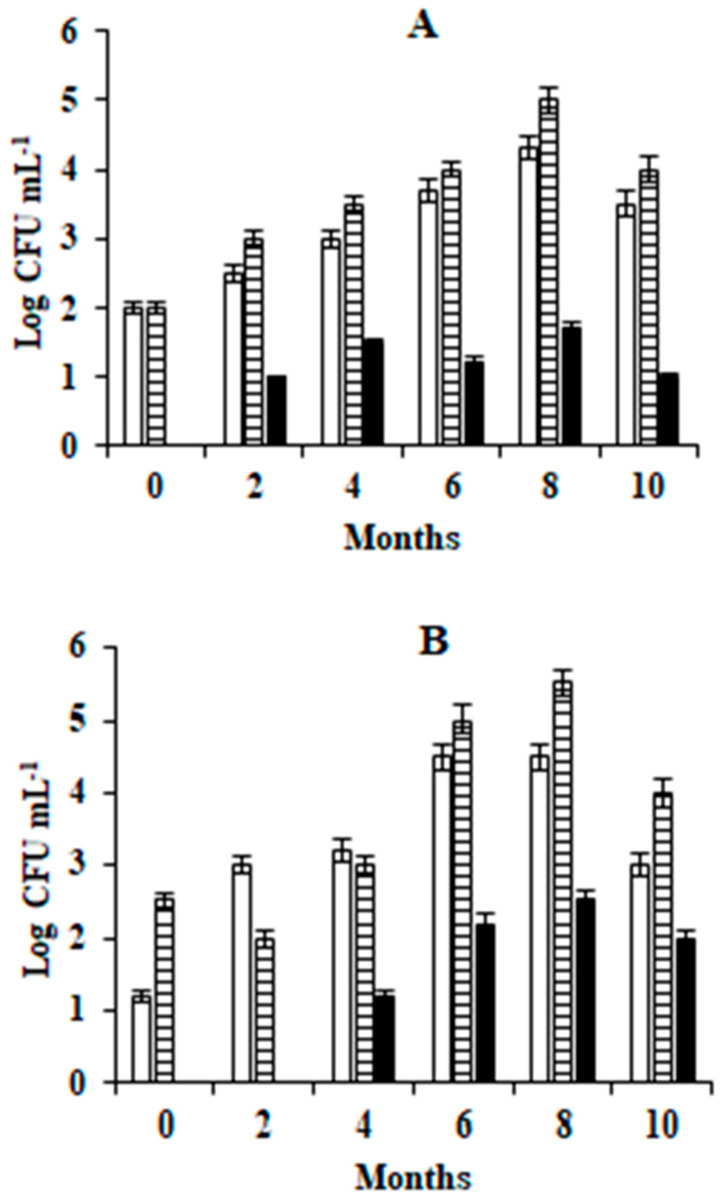
Microbiological analysis of Leccino (**A**) and Taggiasca (**B**) black table olive brines during the fermentation in industrial-scale plastic barrels. (☐, Total aerobic bacteria; ⊟, yeast; ■, mold).

The microbiological analysis of the brine of both varieties of olives revealed the presence of TAB and yeasts during the entire fermentation period in the first year. Mold contamination was observed after two and four months of fermentation in the brines of Leccino and Taggiasca olives, respectively, while LAB and enterobacteria were absent in all the samples analyzed (Figure 3).

Microbiological analysis of the brine samples of both varieties of olives at the end of fermentation revealed the prevalence of the following yeast species: *S. cerevisiae*, *P. manshurica*, *W. anomalus*, *C. boidinii*, and *Z. mrakii*.

#### 3.1.2. Simulation of Yeast Film Formation

Laboratory tests performed with the above yeast species indicated that *W. anomalus* and *P. manshurica* produced films on the MYGP broth medium and olive brine. On the contrary, some strains of *C. boidinii* produced the film only on MYGP broth, while *S. cerevisiae* and *Z. mrakii* did not produce the film on any substrate (Table 1).

**Figure 4:**
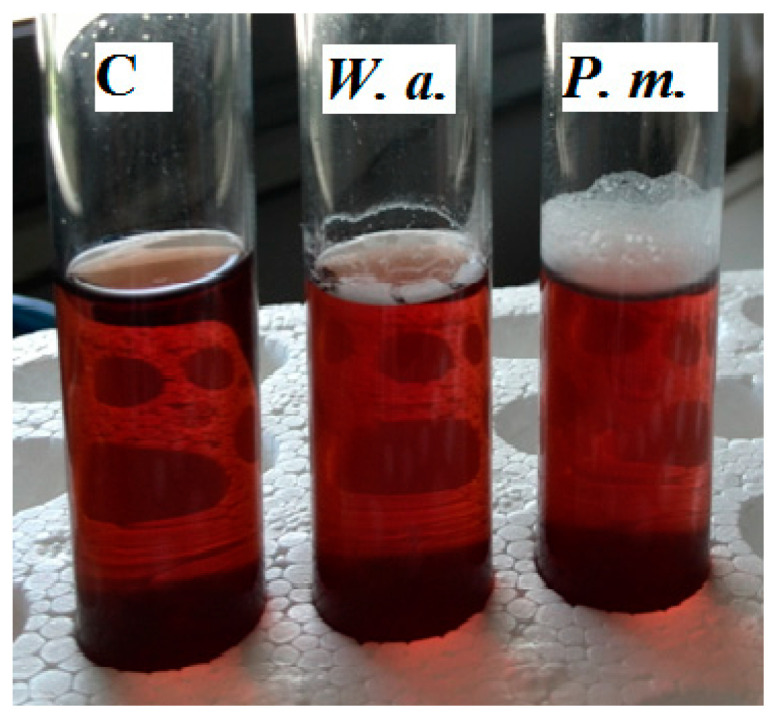
Production of yeast film by *W. anomalus* and *P. manshurica* isolated from the Taggiasca brines and inoculated in the same medium after microfiltration sterilization (C, uninoculated control; *W.a.*, *W. anomalus*; and *P.m.*, *P. manshurica*).

The film produced by the two major yeast-producers represented by *W. anomalus* and *P. manshurica* differed in thickness and extension (Figure 4).

**Figure 5:**
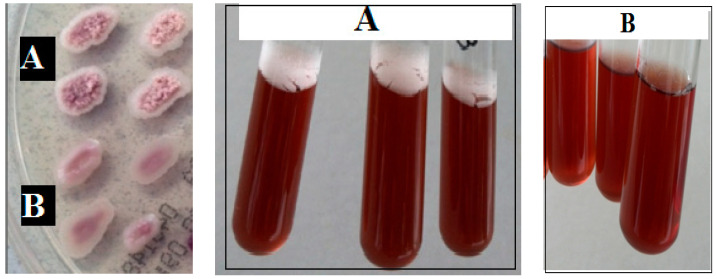
Yeast film from *P. manshurica* producing rough (**A**) or smooth (**B**) colonies inoculated in sterile olive brines.

However, the tests performed with *P. manshurica*, unlike *W. anomalus*, revealed the presence of two physiological variants. The first variant, consisting of *P. manshurica* strains capable of producing rough colonies on the CHROM agar Candida medium, often produced yeast films on the brines surface. The second variant, consisting of strains characterized by smooth colonies on the same medium described before, failed to produce film on the brine surface (Figure 5).

### 3.2. Prevention of Microbial Film Development in Industrial Barrels

The purpose of the second year of trials was to try to prevent the yeast film formation on the surface of the brine through the use of a modified industrial 200 L barrel. The tests performed during the fermentation period showed the complete absence of yeast film contamination of the brines surface from both the olive varieties processed in the modified 200 L industrial barrels. In contrast, the brines of both varieties of olives processed in the unmodified industrial barrels (control) were contaminated by yeast film after four months of fermentation. The extent of the contamination of the control reached the maximum values after eight months of fermentation equal to 13.80% and 11.00%, respectively, for the Leccino and Taggiasca olives (Table 2).

#### 3.2.1. Microbiota Characteristics of Olive Brines from the Two Different Barrel Types

During the fermentation, the brines of Leccino and Taggiasca olives processed in the modified and unmodified barrels showed the presence of TAB and yeasts. In contrast, LAB and enterobacteria were absent in all samples, whereas the molds were present only in the brines of Leccino olives processed in the unmodified barrels (Table 3).

**Figure 6:**
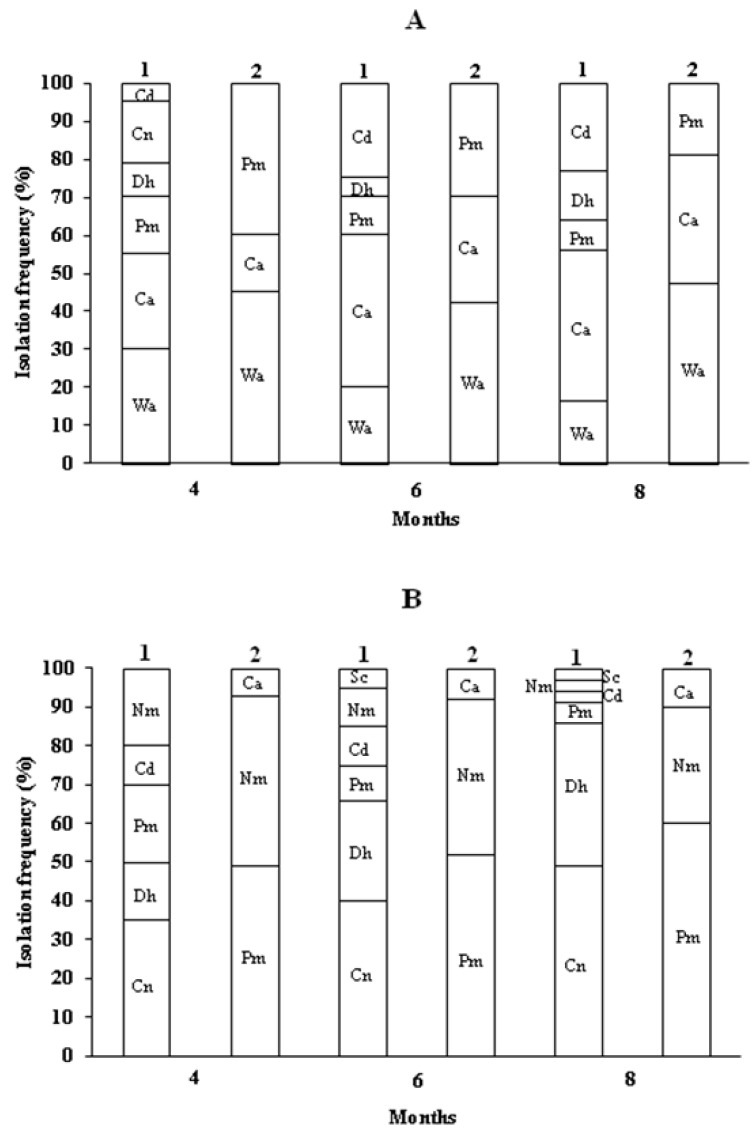
Isolation frequency of yeast species from brines of Leccino (**A**) and Taggiasca (**B**) black olives processed in modified (1) and unmodified (control) (2) industrial plastic barrels. Wa, *W. anomalus*; Ca, *C. adriatica*; Pm, *P. manshurica*; Dh, *D. hansenii*; Cn, *C. norvegica*; Cd, *C. diddensiae*; Nm, *N. molendinolei*; and Sc, *S. cerevisiae.*

However, although the total number of yeasts was similar across all the brines analyzed, the composition of the yeast microbiota varied with the type of barrels used. In the samples of brines of both varieties of olives processed in the modified barrels, more than five species of yeasts were ascertained, whereas no more than three species were identified in the unmodified barrels (Figure 6). The yeast species isolated from the Leccino olive brines processed in the modified barrels were *Candida diddensiae*, *Candida norvegica*, *Debaryomyces hansenii*, *P. manshurica*, *Candida adriatica*, and *W. anomalus*, while those coming from Taggiasca olives were *Nakazawaea molendinolei*, *C. diddensiae*, *P. manshurica*, *D. hansenii*, *C. norvegica*, and *S. cerevisiae*. In contrast, the following species were isolated from the brines taken from the unmodified barrels: *P. manshurica*, *C. adriatica*, and *W. anomalus* for the Leccino variety and *C. adriatica, N. molendinolei*, and *P. manshurica* for the Taggiasca variety (Figure 6).

#### 3.2.2. Physicochemical Characteristics of Olive brines

The values of the physicochemical parameters of the brines of the Leccino and Taggiasca olives, processed in the modified barrels differed significantly from those recorded in the control (unmodified barrel). In detail, the brines of both olive varieties collected from the modified barrels showed values of titratable acidity that tended to be higher than the control during the last four months of fermentation. The same trend was also recorded in the last period of fermentation for the content of total polar phenolic compounds. Significantly higher differences were recorded in the CO_2_ content. In fact, the brines of both varieties of olives from the modified barrels had a CO_2_ concentration that was clearly higher than that of the control, especially in those of the Leccino variety. More specifically, during fermentation, the brines from the modified barrels, compared to the control, showed an increase in CO_2_ between 500 and 750 mg/L for the Leccino variety and from 259 to 559 mg/L for the Taggiasca variety (Table 4).

## 4. Discussion

**Figure A1:**
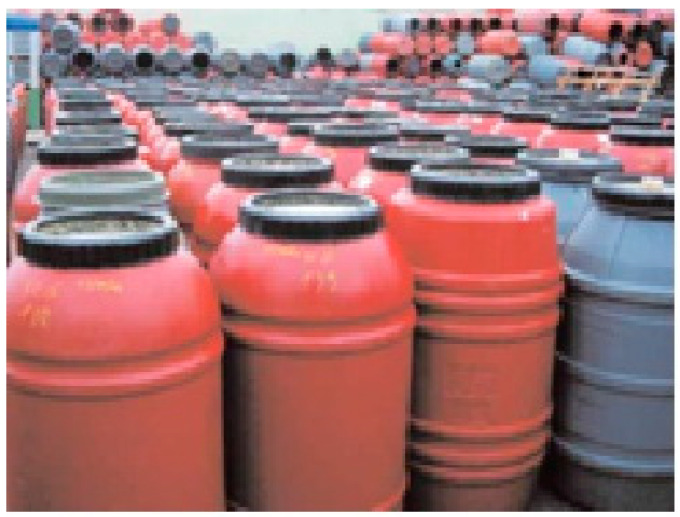
Different types of industrial plastic barrels used in the black table olive processing.

**Figure A2:**
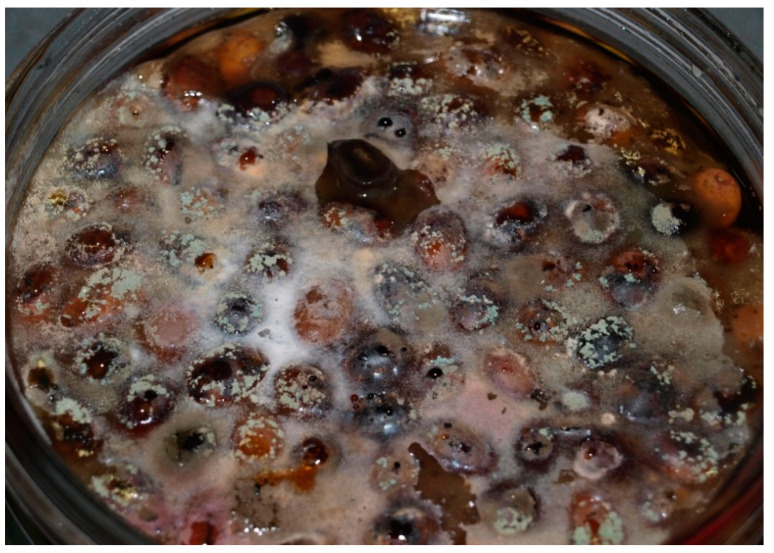
Unmodified industrial barrel (control) with microbial films in the headspace.

**Figure A3:**
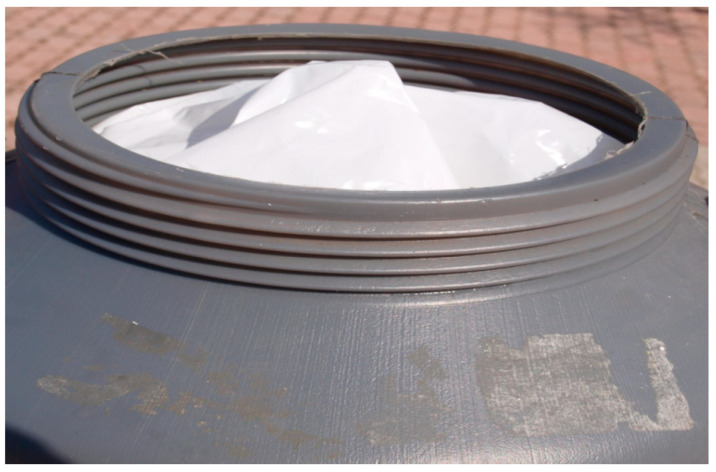
Modified industrial barrel with air-protected headspace.

The use of the plastic barrels is widespread among both small and large processing companies (Figure A1). The barrels are preferred to other technical solutions because are practical to use and, at the same time, are inexpensive since they can be reused in the next production cycle until their expiration date. Plastic barrels perform multiple functions that include moving the olives from one location to another, often more distant, location as happens for products imported from other country, or as containers for pickled olives in which the biochemical processes take place for a period of 4–6 months. At the end of the debittering process, the barrels are still used for the storage of the product until it is opened the point of sale. All these phases often take place with the same containers, and the biochemical processes can be adversely affected by the penetration of the air in the headspace due to the movement of the barrels, changes in temperature during travel, storage place characteristics, and the compromise of the gasket seal. The penetration of they air into the barrel headspace permits the formation of the microbial film, which is often a complex aggregation of microorganisms associated in some cases with an extracellular polymeric polysaccharide matrix (Figure A2) [12]. The yeast film is represented by a harmful surface layer of cells of some species of oxidative yeasts which reproduce rapidly in the surface of the brine richest in oxygen. Usually, the metabolism of yeasts can follow two pathways. One is the respiration pathway, which takes place in the presence of oxygen and releases water and CO_2_ as final compounds. The second is the fermentation pathway, which takes place anaerobically and which generates CO_2_ and many intermediate metabolites that are useful for the organoleptic characteristics of pickled olives [25,26]. For the successful outcome of the full transformation process, it is necessary to maintain the anaerobic environment of the brines stored in the barrels during all the working phases describe before. The tests performed during the first year of investigation revealed the frequent development of microbial films on brines, regardless of the variety of olive. The appearance of the film in the barrels of both varieties of olives (Figure 1) recorded in the eighth month of conservation (greater than 18%), seem to be related to the increase in temperature during the summer months. The appearance of the microbial film after the first two months of fermentation in both olive varieties can be explained by considering various factors. These include a low winter temperature that is not appropriate to the growth of yeasts and minimal temperature variations between day and night, in which brine dilatation permits the penetration of external air into the barrels and the higher concentration of CO_2_ in the brine coming from the initial fermentation phase [27]. Microbiological analyses showed the presence of aerobic bacteria, yeasts and molds, but not enterobacteria and LAB (Figure 3). The absence of LAB can be attributed to the high concentration of NaCl in the brine and its acidification with citric acid [8,9,10]. However, it should be noted that the direct and SEM observations revealed that the composition of the microbial film differed from that of the microbiota of the underlying brine, since the film was often comprised of yeasts (Figure 2). The main yeasts were *W. anomalus* and *P. manshurica*. However, the film produced in the brine by the latter species differed from that produced by *W. anomalus* in that it was quite thin and tended to adhere to abiotic surfaces, including the glass (Figure 4). The two variants of *P. manshurica* consisted of strains that often formed films and those that seldom did (Figure 5). This is the first description of these *P. manshurica* variants in the brines of Leccino and Taggiasca olives processed at industrial-scale. The different morphology of the colonies produced by the two different types of yeast could be a useful marker in the selection of *P. manshurica* starter that seldom produces films on brines exposed to air. The tests carried out at the processing company during the first year of trials showed that if the microbial films were not promptly removed from the surface of the brines and the contaminated barrels refilled with new brine, the probability of having to discard the contents because of spoilage could be very high. In fact, without technical interventions capable of preventing the penetration of air into the barrels, serious economic losses could be incurred by the processing company. The results obtained from the tests carried out during the second year of tests demonstrated that the modified barrels, unlike the unmodified control barrels, were very effective in preventing yeast film formation on brines. Unlike the control that was characterized a barrel contamination rate exceeding 10%, all the brines of both varieties of olives processed in the modified barrels were free of yeast films (Table 2). Compared to the control, the microbiota of all the brine samples taken from the modified barrels included the absence of mold, a smaller number of TAB (Table 3), and a greater number of yeast species (Figure 6). The absence of mold and the lower concentration of TAB can be linked to the better anaerobic conditions of these brines, which were richer in CO_2_ (Table 4). On the contrary, the total yeast count did not change compared to the control (Table 3), since the anaerobic conditions of these brines result in balance between the mainly oxidative yeast species with those characterized by a fermentative metabolism (Figure 6). The physicochemical characteristics of the brines taken from the modified barrels (Table 4) differed from those of the control mainly by their higher CO_2_ content, which increased the titratable acidity and simultaneously allowed the maintenance of pH values within acceptable limits for proper fermentation [28]. During the entire fermentation period, the free CO_2_, balanced by that dissolved in the brine, due to the formation of a slight pressure inside the closed bags of the modified barrels (Figure A3), prevented the penetration of the external air due to the various causes described earlier. The parameters related to product quality also improved when the two varieties of olives were processed using modified barrels.

## 5. Conclusions

The tests performed in the first year of trials showed that more than 18% of black table olive brines processed in industrial scale plastic barrels were contaminated with microbial films that were detrimental to the product. The appearance of microbial films occurred after the first two months of fermentation and developed most frequently between six and eight months of fermentation. If films were not manually removed from the surface of the brines during their initial development phase, the content of the contaminated barrels could be seriously compromised in a short time, with serious economic losses for the processing company. The tests carried out in the second year indicated that the contamination of brines with microbial films can be solved using a novel design of barrel that protects the air headspace. The novel barrel represents only a small modification of the conventional industrial plastic barrels. Compared to the control, brines of both varieties of olives processed in the modified barrels displayed a higher titratable acidity and a higher concentration of CO_2_ that inhibited the growth of the film-producing yeasts. Unlike the control, which was contaminated by yeast films in 11%−14% of the barrels, brines from modified barrels were free of yeast films.

## Figures and Tables

**Table 1:** Yeast film production in vitro by the predominant species isolated from brines after 10 months of fermentation in industrial barrels.

Yeast	MYGP Broth	Table Olive Brines
*Saccharomyces cerevisiae* (L)	-	-
*Saccharomyces cerevisiae* (T)	-	-
*Pichia manshurica* 2051 (L)	++	++
*Pichia manshurica* 2081 (T)	++	++
*Wickerhamomyces anomalus* (L)	+++	+++
*Wickerhamomyces anomalus* (T)	+++	+++
*Candida boidinii* (L)	+	-
*Candida boidinii* (T)	-	-
*Zygosaccharomyces mrakii* (L)	-	-
*Zygosaccharomyces mrakii* (T)	-	-

-, absence of surface film; +, low film production; ++, medium film production; and +++, high film production; L, Leccino; and T, Taggiasca.

**Table 2:** Yeast film contamination (%) of brines stored in modified and unmodified industrial barrels during fermentation.

Treatment	2 ^1^	4	6	8
Leccino variety:				
modified barrel	0.00	0.00	0.00	0.00
unmodified barrel	0.00	2.00 ± 0.73	8.70 ± 1.92	13.80 ± 2.21
Taggiasca variety:				
modified barrel	0.00	0.00	0.00	0.00
unmodified barrel	0.00	1.50 ± 0.47	6.30 ± 1.11	11.00 ± 2.40

^1^ Months of fermentation.

**Table 3:** Microbiological analysis of the brine from Leccino and Taggiasca black olives processed in two different types of industrial barrels.

Table Olives	4 Months				6 Months				8 Months			
	LAB ^1^	TAB ^2^	Yeast	Mold	LAB	TAB	Yeast	Mold	LAB	TAB	Yeast	Mold
Leccino variety:												
modified barrel	0	4.34 ± 0.18 ^b^	5.29 ± 0.37	0	0	4.47± 0.25 ^ab^	4.73 ± 0.21	0	0	4.78± 0.30 ^ab^	3.70 ± 0.18	0
unmodified barrel	0	6.77 ± 0.32 ^a^	5.76 ± 0.42	4.18 ± 0.1	0	5.70 ± 0.30 ^a^	4.58 ± 0.60	3.20 ± 0.18	0	5.54 ± 0.50 ^a^	4.40 ± 0.32	2.70 ± 0.20
Taggiasca variety:												
modified barrel	0	3.60 ± 0.15 ^b^	5.81 ± 0.15	0	0	3.20 ± 0.18 ^b^	6.32 ± 0.50	0	0	3.01 ± 0.12 ^b^	5.67 ± 0.50	0
unmodified barrel	0	5.10 ± 0.20 ^a^	5.36 ± 0.40	0	0	4.60± 0.35 ^ab^	5.57 ± 0.46	0	0	4.50± 0.35 ^ab^	5.09 ± 0.24	0

^1^ LAB, lactic acid bacteria. ^2^ TAB, Total aerobic bacteria. Data are reported as mean and standard deviation of log CFU/mL. Different letters in the same column indicate significant differences by Duncan’s multiple tests (*p* < 0.05).

**Table 4:** Chemical analysis of brines from Leccino and Taggiasca black olives processed in two different types of industrial barrels.

Table Olives	4 Months					6 Months					8 Months				
	pH	Titratable Acidity ^1^	Total Phenols ^2^	CO_2_^3^	Δ ^4^	pH	Titratable Acidity	Total Phenols	CO_2_	Δ	pH	Titratable Acidity	Total Phenols	CO_2_	Δ
Leccino variety:															
modified barrel	4.48 ± 0.13	5.04 ± 0.14 ^ab^	1.16 ± 0.11	1598± 4.54 ^a^	719	4.36 ± 0.13	5.34 ± 0.34 ^ab^	1.02 ± 0.01 ^ab^	1395 ± 5.65 ^a^	750	4.29 ± 0.11	4.97 ± 0.16 ^b^	1.40 ± 0.08 ^ab^	959 ± 1.23 ^a^	500
unmodified barrel	4.59 ± 0.11	3.48 ± 0.13 ^b^	1.21 ± 0.09	879 ± 3.01 ^c^	-	4.50 ± 0.17	4.64 ± 0.22 ^b^	0.71 ± 0.01 ^b^	645 ± 4.32 ^c^	-	4.50 ± 0.21	3.83 ± 0.17 ^c^	1.10 ± 0.07 ^b^	459 ± 2.01 ^b^	-
Taggiasca variety:															
modified barrel	4.32 ± 0.12	6.73 ± 0.21 ^a^	0.99 ± 0.06	1118 ± 2.3 ^b^	259	4.26 ± 0.11	8.10 ± 0.43 ^a^	1.53 ± 0.03 ^a^	1124 ± 4.12 ^b^	559	4.28 ± 0.09	8.24 ± 0.54 ^a^	1.80 ± 0.05 ^a^	999 ± 1.02 ^a^	530
unmodified barrel	4.41 ± 0.09	5.90 ± 0.19 ^ab^	0.92 ± 0.05	859 ± 1.24 ^c^	-	4.32 ± 0.03	6.99 ± 0.54 ^ab^	1.00 ± 0.04 ^ab^	565 ± 2.12 ^c^	-	4.35 ± 0.02	7.11 ± 0.20 ^ab^	1.20 ± 0.02 ^b^	469 ± 1.21 ^b^	-

^1^ Titratable acidity refers to g of lactic acid per L of brine. ^2^ Total phenols refers to mg of caffeic acid equivalent per mL of brine. ^3^ The free CO_2_ content was expressed as mg CO_2_ per L of brine. ^4^ Increase in CO_2_ concentration in the brines stored in the modified barrels compared to the unmodified ones. Different letters in the same column indicate significant difference by Duncan’s multiple tests (*p* < 0.05).
